# The role of total cell-free DNA in predicting outcomes among trauma patients in the intensive care unit: a systematic review

**DOI:** 10.1186/s13054-016-1578-9

**Published:** 2017-01-24

**Authors:** Mikail Gögenur, Jakob Burcharth, Ismail Gögenur

**Affiliations:** Center for Surgical Science, Department of Gastrointestinal Surgery, Zealand University Hospital, Lykkebækvej 1, 4600 Køge, Denmark

**Keywords:** cfDNA, mtDNA, nDNA, Trauma, Intensive care unit

## Abstract

**Background:**

Cell-free DNA has been proposed as a means of predicting complications among severely injured patients. The purpose of this systematic review was to assess whether cell-free DNA was useful as a prognostic biomarker for outcomes in trauma patients in the intensive care unit.

**Methods:**

We searched Pubmed, Embase, Scopus and the Cochrane Central Register for Controlled Trials and reference lists of relevant articles for studies that assessed the prognostic value of cell-free DNA detection in trauma patients in the intensive care unit. Outcomes of interest included survival, posttraumatic complications and severity of trauma. Due to considerable heterogeneity between the included studies, a checklist was formed to assess quality of cell-free DNA measurement.

**Results:**

A total of 14 observational studies, including 904 patients, were eligible for analysis. Ten studies were designed as prospective cohort studies; three studies included selected patients from a cohort while one study was of a retrospective design. We found a significant correlation between higher values of cell-free DNA and higher mortality. This significant correlation was evident as early as on intensive care unit admission. Likewise, cell-free DNA predicted the severity of trauma and posttraumatic complications in a majority of patients.

**Conclusion:**

The amount of cell-free DNA can function as a prognostic tool for mortality and to a lesser extent severity of trauma and posttraumatic complications. Standardizing cell-free DNA measurement is paramount to ensure further research in cell-free DNA as a prognostic tool.

**Electronic supplementary material:**

The online version of this article (doi:10.1186/s13054-016-1578-9) contains supplementary material, which is available to authorized users.

## Background

In recent years cell-free DNA (cfDNA) has become increasingly used as a clinical and noninvasive biomarker in the fields of cancer [1–3], pre-natal diagnostics [4], organ transplantation [5], and in several emergency conditions [6–8]. cfDNA, defined as extracellular DNA circulating freely in the blood, can be further subcategorized to circulating mitochondrial DNA (mtDNA) and circulating nuclear DNA (nDNA). Within cancer research, cfDNA has been proposed to have the ability to act as a noninvasive biopsy of the tumor (i.e., liquid-biopsy) [9] and as a prognostic marker for clinical outcomes such as disease burden [3], progression-free survival [10], and overall survival [11].

In patients admitted to an intensive care unit (ICU) as a result of trauma, cfDNA has received increasing attention under the hypothesis that cfDNA originates from cell death [12, 13] and could correlate with the severity of trauma with prognostic and predictive abilities. Preliminary reports have confirmed that the amount of cfDNA correlates inversely to mortality [14], trauma severity [15], and post-traumatic complications [16]. Due to the short half-life of cfDNA [17], it is suitable as a marker of the patient’s condition in the immediate emergency phase. mtDNA has also been increasingly investigated in trauma patients in recent years and it has been argued that mtDNA could be considered a damage-associated molecular pattern (DAMP). It is well established that circulating DAMPs lead to an increase in the innate immune response [18], possible leading to a systematic inflammatory response syndrome (SIRS) [19].

The use of cfDNA as a predictive marker of clinical outcome have not been systematically analyzed. The aim of this study was to review the literature on cfDNA as a predictive marker of clinical outcomes as measured in trauma patients in ICUs.

## Methods

This systematic review was conducted according to the Preferred Reporting Items for Systematic reviews and Meta-Analyses (PRISMA) statement [20].

### Eligibility criteria

The inclusion criteria for studies in this review were cohort human studies that investigated levels of cfDNA in plasma or serum in trauma patients aged ≥18 years in the ICU. Trauma was defined as all grades of trauma ranging from minor to severe trauma, including isolated traumatic brain injury (TBI) that resulted in ICU admission. Studies exclusively evaluating circulating RNA as well as studies conducted outside the ICUs were excluded. A thorough assessment of the quality of DNA sampling and processing was conducted for all included studies using previous definitions [21] (see Additional file 1). We included studies that analyzed DNA by specific sequencing (beta-globin, GAPDH, NADH dehydrogenase) or fluorescent methods. We only included published studies and only studies published in the English language.

### Study search

A computerized comprehensive search strategy was conducted using four databases (PubMed, EMBASE, SCOPUS, and the Cochrane Central Register for Controlled Trials) from January 1974 to January 2016. The search was performed on 20 January 2016. The following literature search was used in PubMed: “(circulating cell free dna) OR cfdna) OR circulating nucleic acids) OR cell free mitochondrial DNA) OR nDNA) AND (injury) OR trauma) OR stress) OR surgery) OR intensive care unit) OR perioperative) OR postoperative) OR intraoperative) OR preoperative)”.

The literature search was adapted from the PubMed literature search to EMBASE, SCOPUS, and the Cochrane Central Register for Controlled Trials. We supplemented the structured literature search with searching of the reference lists from the included articles in order to find additional eligible studies.

### Study selection

The Cochrane systematic review tool Covidence.org was used in the screening process. Two reviewers (MG and JB) independently screened titles and abstracts until the full-text articles were found. Two authors (MG and JB) independently assessed the full-text articles. Whenever different opinions emerged a third author (IG) was included in the discussion until consensus was reached.

### Data collection and data items

All included articles were assessed for the following information: publication details, study method, patient details, specific time points of cfDNA measurements, mortality, post-traumatic complications, and trauma severity. This data was collected using a data sheet.

### Risk of bias of individual studies

Risk of bias assessment was performed by two authors (MG and JB) using an adapted version of the Scottish Intercollegiate Guidelines Network (SIGN) (http://www.sign.ac.uk/methodology/checklists.html) checklist for cohort studies [22]. The SIGN checklist evaluates selection bias, performance bias, attrition bias, detection bias, and statistical analysis. All studies were assigned up to 8 points if they met the SIGN checklist criteria. Studies with 8 points were considered to be high quality (HQ) studies with little or no risk of bias, while studies between 6 and 7 and 0 and 5 were considered acceptable quality (AQ) with an associated risk of bias and unacceptable quality with a high risk of bias, respectively.

One point was given for every checkpoint met, while studies with insufficient information regarding a checkpoint were given 0 points. Checkpoints not applicable for certain studies were given 1 point as it was not considered a shortcoming of the quality of the study.

Different techniques and methods have been developed over the past years to process and analyze cfDNA. This was taken into consideration by a thorough assessment of DNA sampling and processing in all included studies using previously described methods [21] on the quality of cfDNA processing and analyses (see Additional file 1). We adopted these recommendations of cfDNA processing into a list of checkpoints for each study. Checkpoints considered the type of medium for analysis (plasma or serum), the amount and speed of centrifugation, type of sample tube, freezing of samples and under which conditions (–20 or –80 °C), and finally if blood had been processed before 4 hours. The results of these assessments were collected on the same data sheet as outcomes. Greater than 5 points were deemed as high-quality circulating DNA processing, 3–4 points were deemed as acceptable quality, while 0–2 points were considered unacceptable quality. The quality of the DNA processing was taken into account in evaluating the robustness of study conclusions.

## Results

### Study selection

**Fig. 1:**
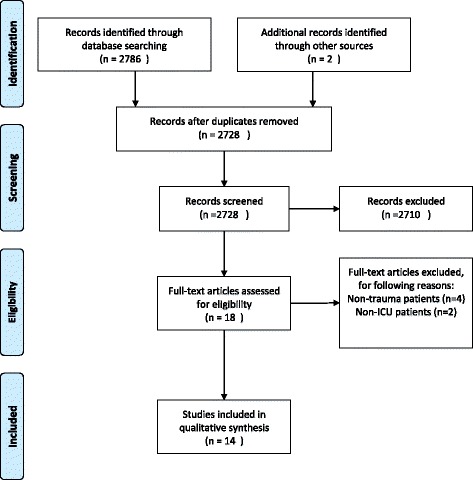
Flow diagram. PRISMA flow diagram of included studies in qualitative synthesis. *ICU* intensive care unit

The search identified 2728 potential studies (see Fig. 1), where 2710 studies by title and abstract screening were deemed irrelevant. Of the remaining 18 studies, six studies were excluded for the following reason: four studies were based on nontrauma patients [23–26] and two studies included both ICU and nonICU patients [27, 28]. A total of 15 studies from 14 references were included in this systematic review of which two studies were added after examining the included studies’ references lists (see Fig. 1). Ten studies were designed as prospective cohort studies, three studies included selected patients from a cohort, while one study included retrospective patients.

### Study characteristics

The main characteristics and cfDNA processing score (see Additional file 2 for information regarding specific points) of the included studies are summarized in Table 1. A total of 904 patients were included in the period 1996–2013 with a sample size range of 25–188. One reference presented results from two studies leading to two separate DNA processing and risk of bias assessments [29]. Ten studies included nonspecific trauma patients while five studies included patients with TBI with or without extracranial trauma [16, 30–36]. Nine of the nonspecific trauma studies based severity of trauma on the Injury Severity Score (ISS) [14, 15, 29, 34–38], while one used both the Acute Physiology and Chronic Health Evaluation (APACHE) II and Sepsis-related Organ Failure Assessment (SOFA) score [39]. All TBI studies used the Glasgow Coma Scale (GCS) to classify the effects of the trauma while using either the abbreviated injury scale (AIS), ISS, or APACHE II to describe the severity of trauma. The DNA processing score ranged from 2–5 (Table 1). All studies measured cfDNA on ICU admission, while eight studies also measured cfDNA at different time points during the admission period.

**Table 1:** Study characteristics

Author	Method	Patients, *n*	Age, years	Sex male, *n* (%)	Time of measurement	Severity score (*n*) [range]	DNA measurement score^a^
Nonspecific trauma							
Ren et al. (2013) [37]	Cohort study	56	38 ± 14	47 (83.9%)	1–6 h, 24–36 h, 60–90 h	ISS, minor: 4.8 ± 1.7 (16) ISS, moderate: 11.1 ± 1.9 (19) ISS, severe: 33.7 ± 20.0 (21)	4
Margraf et al. (2008) [15]	Prospective cohort	37	45 ± 21.3	24 (64.9%)	ICU admission, 1–10 days	ISS, 31.6 ± 11 [16–50]	5
Lo et al. (2000) [14]	Retrospective study	84			ICU admission	ISS, minor/moderate: <16 (47) ISS, major: ≥16 (37)	3
Wijeratne et al. (2004) [39]	Cohort study	96	63 (25–85)	69 (73.4%)	ICU admission	?	5
Lam et al. (2003) [29]	Observational study	25	38	24 (63.2%)	Study 1: ICU admission, every 20 min for 180 min Study 2: ICU admission every day for 28 days	ISS, minor: <9 (4) ISS, moderate: 8–15 (10) ISS, severe:16–24 (7) ISS, very severe: >25 (4)	3
McIlroy et al. (2014) [43]	Prospective cohort and healthy controls	35	38 (29–48)	25 (71.4%)	Preoperative, immediately postoperative, 7 h postoperative, 24 h postoperative, 3 days postoperative, 5 days postoperative	Median ISS: 14 [9-22]	4
Yamanouchi et al. (2013) [34]	Prospective study	37	56 (35–70)	26 (70.2%)	ICU admission, days 1–2 and day 4	AIS >3 SOFA score (day 1) 2 [2-4] APACHE II score (day 1) 11 [6-15]	2
Lam et al. (2004) [36]	Prospective cohort and healthy controls	38	NA	NA	ICU admission	ISS <16 (28) ISS >16 (10)	4
Gu et al. (2013) [35]	Prospective cohort and healthy controls	86	45.5 (28.75–57.25)	61 (70.1%)	ICU admission	SIRS absent ISS 14 [9.75-18.25] (50) SIRS present ISS 22 [18-29] (36) SIRS present APACHE II 11.5 [8-16] SIRS absent APACHE II 9 [6-11]	4
Traumatic brain injury							
Macher et al. (2012) [30]	Prospective cohort and healthy controls	65	38.18 ± 2.02	56 (86%)	ICU admission, 24 h, 48 h, 72 h and 96 h postoperative	GCS: 7 [3-9] ISS: 36 [9-75] APACHE II: 19 [5-34]	2
Shaked et al. (2014) [31]	Selected patients from a cohort	28	49 (18–91)	23 (82.1%)	ICU admission	GCS ≥14 (14) GCS ≤13 (14) AIS 0–2 (10) AIS 3–5 (18)	
Yurgel et al. (2007) [32]	Selected patients from a cohort	41	34 (18–64)	41 (100%)	Study entry, 24 h later	GCS survivors: 6.5 GCS nonsurvivors: 5 APACHE II survivors: 12.5 APACHE II nonsurvivors: 18.3	2
Filho et al. (2014) [33]	Prospective cohort and healthy controls	188	34.8 (13.9)	165 (88%)	ICU admission	GCS survivors: 6.3 GCS nonsurvivors: 5.2	4
Wang et al. (2014) [16]	Prospective cohort	88	36 (20–53.75)	55 (62.5%)	ICU admission, 4 days, 7 days	GCS: 15 [13-15] ISS: 16 [11-20]	4

Values are given as mean ± SD or median (interquartile range) unless indicated otherwise

*AIS* abbreviated injury scale, *APACHE* Acute Physiology and Chronic Health Evaluation, *GCS* Glasgow Coma Scale, *ICU* intensive care unit, *ISS* Injury Severity Score, *NA* not available, *SOFA* Sepsis-related Organ Failure Assessment, *SIRS* systemic inflammatory response syndrome

^a^Every study was assessed for cfDNA sampling and analysis: 1 point was given if circulating DNA was analyzed in plasma; 1 point was given if blood was collected in either an EDTA tube or cell-free DNA tube; 1 point was given if blood was processed before 4 h; 1 point was given if blood was centrifuged one or more times; 1 point was given if blood was frozen at –80 °C or –20 °C depending on whether cfDNA analysis was based on specific sequence or cfDNA quantification, respectively

The patient population differed to some extent between the studies. Ten studies included all trauma patients in the ICU [14, 15, 29, 34–39], while five studies required patients to be diagnosed with TBI [16, 30–33]. One study only included male patients [32]. Treatment strategies during ICU admission were not described, which mean that different treatments might have been used. There is a risk of bacterial DNA contamination in DNA assessment methods, but only one study reported on bacterial DNA analyses [38].

### Risk of bias within studies

Risk of bias assessments are presented in Additional file 3. According to the adapted version of the SIGN checklist (Additional file 4), one study was rated as a high-quality study [16] while 13 studies were rated as being of acceptable quality and one was rated as of unacceptable quality. Eleven of 15 studies had control groups [14, 16, 30–33, 35–39]; 12 studies failed to deliver adjusted confidence intervals [14, 15, 29–31, 34, 36–39], and a total of five studies were deemed to have a high risk of selection bias as a consequence of a retrospective design [14], failure to provide exclusion criteria [39], and nonconsecutive patient inclusion [29, 32]. A possibility of detection bias was assessed in 11 of 15 groups as a consequence of these not providing information regarding the blinding of essential risk factors such as severity of trauma or clinical outcome [14, 15, 30–32, 34–39].

Two studies were considered as high-quality studies based on DNA processing [15, 39] while nine were deemed of acceptable quality [14, 16, 29, 33, 35–38] and three presented with an unacceptable quality of DNA processing [30, 32, 34]. One study was not assessed as no information regarding blood sampling and processing was available [31]. Twelve of 15 studies used plasma [14–16, 29, 32–39]. All studies centrifuged blood samples prior to freezing. None of the TBI studies stored the samples at –80 °C while 9 of 10 nonspecific trauma studies did [15, 29, 34–39].

## Results of individual studies

### Severity of trauma and post-traumatic complications

The main outcomes are presented in Table 2. In a study of 56 patients, no correlation was found between severity of trauma scored with ISS and levels of cfDNA [37]; however, four other studies found a significant correlation between cfDNA and ISS score. Patients diagnosed with acute lung injury (ALI) or acute respiratory distress syndrome (ARDS) had significantly higher cfDNA compared to patients without these diagnoses [14]. This corresponds to a study which proved that repeatedly measured levels of cfDNA 1 h, 2 h, and 3 h after ICU admission in 25 patients at all time points were significantly correlated with a higher degree of injury [29]. ISS scores were also correlated to cfDNA in a prospective study from 2008 on 37 patients [15] which investigated cfDNA originating from neutrophils using a fluorescent method, which was able to identify three different types of cfDNA kinetics. Type 1 kinetics were characterized by an initial value of cfDNA below 800 ng/mL followed by a rapid decrease. Type 2 kinetics were characterized by initial values above 800 ng/mL followed by a rapid decrease. Type 3 kinetics of cfDNA was characterized by high initial values above 800 ng/mL and a rapid decrease on days 1–5 followed by a significant increase of cfDNA on days 7–10 compared to type 1 and 2 kinetics. Type 3 kinetics were also associated with a higher ISS score, but not significantly. A significant association between type 3 kinetics and sepsis was found.

**Table 2:** CfDNA’s ability to predict outcomes

Article/outcomes	Survival	Complications	Severity score	Cut-off
Nonspecific trauma				
Ren et al. [37]	NA	Not able to predict secondary infection	No significant correlation between cfDNA and ISS	700 copies/L plasma sensitivity: 41.1% specificity: 96.7% PPV: 95.85% NPV: 46.8%
Margraf et al. [15]	Lower levels of cfDNA had a NPV of 100% for MOF and death	Higher levels of cfDNA on admission and on days 7–10 was significantly associated with sepsis	Higher levels of cfDNA correlated with higher ISS scores.	NA
Lo et al. [14]	All patients undergoing death had significantly higher cfDNA than survivors	ALI and ARDS patients had significantly higher cfDNA than patients without this diagnosis	Higher levels of cfDNA correlated with higher ISS scores.	232.719 KE/L had a sensitivity and specificity for death of 78% and 82%, respectively
Wijeratne et al. [39]	2.3-fold higher cfDNA in nonsurvivors than in survivors (no *p* value)	Ventilated patients had significantly higher cfDNA values than nonventilated patients	Higher levels of cfDNA correlated with higher SOFA scores but not with APACHE II	6.109 GE/mL sensitivity and specificity at 85% and 80%, respectively, for death
Lam et al. [29]	Study 1: NA Study 2: NA	Study 1: statistical difference between patients with OF and nonOF patients Study 2: on days 2, 3, 4, and 5 patients with MODS had significantly higher cfDNA that nonMODS patients	Study 1: At 1 h, 2 h, 3 h cfDNA significantly higher in patients with severe injury compared to less severely injured patients. Study 2: NA	NA
McIlroy et al. [43]	Not powered	No correlation between levels of mtDNA or nDNA in relation to SIRS or MOF	Not powered	Not powered
Yamanouchi et al. [34]	Significantly higher mtDNA in nonsurvivors compared to survivors (2 patients died)	No correlation with SOFA or APACHE II scores	High mtDNA significantly correlated with high ISS score (*p* < 0.05)	NA
Lam et al. [36]	Significantly higher mtDNA in nonsurvivors compared to survivors (2 patients died)	NA	Patients with severe injury (ISS >16) had significantly higher mtDNA than patients with minor/moderate injury (ISS < 16). Similar results for nDNA	NA
Gu et al. [35]	NA	mtDNA on ICU admission able to predict SIRS (*p* < 0.001)	mtDNA significantly correlated to high APACHE II score(*p* = 0.034) and ISS score (*p* < 0.001)	Cut-off value of mtDNA 1.3185 μg/ml with a sensitivity of 67% and specificity of 75%
Traumatic brain injury				
Macher et al. [30]	Significantly higher decrease of cfDNA from 0 to 24 h in survivors than in nonsurvivors	Patients with high GCS score (11–15) had significantly lower serum DNA levels at admission and 24 h than severe TBI patients	Patients with high APACHE II (<15) score and ISS had significantly higher cfDNA that patients with lower APACHE II score and ISS at 24 h	A cut-off ratio of 1.95 had a sensitivity and specificity of 70% and 66%, respectively
Shaked et al. [31]	Significantly higher levels of cfDNA in nonsurvivors than in survivors	Significantly higher cfDNA in patients with GOS score ≤4 vs patients with GOS score 5		A cut-off ratio of 700 ng/ml had a sensitivity and specificity of 82% and 59% to predict GOS <5
Yurgel et al. [32]	Significantly higher levels of cfDNA at 24 h in nonsurvivors than in survivors		No difference in cfDNA between isolated TBI and TBI with extracranial injuries	77,883,5 KE/L at 24 h with a sensitivity and specificity of 67% and 76% for mortality
Filho et al. [33]	Significantly higher levels of cfDNA in nonsurvivors than in survivors	High cfDNA levels on admission siginificantly associated with death	Significantly higher cfDNA in patients with lower GCS score compared with patients with higher GCS score	171.381 KE/L plasma sensitivity of 43%, specificity of 90%
Wang et al. [16]	nDNA had significantly higher levels on days 1, 4, and 7 in patients with poor outcome compared to patients with good outcome	High nDNA significantly correlated with low GCS	High nDNA significantly correlated with high ISS score	72.95 ng/ml sensitivity of 87.5%, specificity of 86.2%

*AIS* abbreviated injury scale, *ALI* acute lung injury, *APACHE* Acute Physiology and Chronic Health Evaluation, *ARDS* acute respiratory distress syndrome, *cfDNA* cell-free DNA, *GCS* Glasgow Coma Scale, *GE* genome equivalent, *GOS* Glasgow outcome scale, *ICU* intensive care unit, *ISS* Injury Severity Score, *KE* kilo equivalent, *MODS* multiple organ dysfunction syndrome, *MOF* multi-organ failure, *mtDNA* circulating mitochondrial DNA, *NA* not available, *nDNA* circulating nuclear DNA, *NPV* negative predictive value, *OF* organ failure, *PPV* positive predictive value, *SOFA* Sepsis-related Organ Failure Assessment, *SIRS* systemic inflammatory response syndrome, *TBI* traumatic brain injury

SOFA and APACHE II scores were investigated in a study on 96 patients which found that higher levels of cfDNA significantly correlated with higher SOFA scores but not with APACHE II score [39]. A study measuring mtDNA in trauma patients found a significant correlation between mtDNA levels in the plasma and APACHE II and ISS scores [35]. Similar results were found in two other studies [34, 36], but one study did not find an association between mtDNA levels and APACHE II score [34].

In a study on patients with severe TBI, high APACHE II and ISS scores were significantly correlated with higher levels of cfDNA 24 h after ICU admission. This study also showed that patients with high GCS score (11–15) had significantly lower cfDNA at both ICU admission and after 24 h [30], which was confirmed in a similar study [33]. However, a significant correlation between patients with isolated TBI and patients with extracranial injuries was not found for cfDNA levels in 41 male patients with TBI [32].

Regarding nDNA, similar results have been presented where patients with high nDNA had lower GCS scores and significantly higher ISS scores [16]. However, one study, reporting on both nDNA and mtDNA, found no correlation between levels of nDNA and mtDNA in relation to SIRS or multi-organ failure (MOF) [38].

### Survival

Survival data in relation to cfDNA was presented in ten studies. One study reported not being powered to present survival data [38], while three references did not present survival data analyzed in relation to cfDNA levels [29, 35, 37]. In the earliest study measuring cfDNA in a retrospective population of trauma patients, it was found that all patients who died in the ICU had significantly higher cfDNA than survivors [14]. Likewise, it has been reported that nonsurvivors had a 2.3-fold higher cfDNA level than survivors [39]. In a prospective cohort of 37 patients, lower cfDNA levels at ICU admission had a negative predictive value (NPV) of 100% in relation to death [15]. In another study, survivors presented significantly lower cfDNA levels on ICU admission and 24 h after, but not 48 h after, ICU admission [30]. Other authors only found this significant correlation at 24 h after ICU admission [31–33]. Two of the studies measuring mtDNA presented survival data, both showing nonsurviving patients with significantly higher mtDNA than survivors [34, 36]. Finally, in a prospective study of 88 patients where nDNA was measured at ICU admission and on days 4 and 7, patients with a fatal outcome had significantly higher nDNA at all time points [16].

## Discussion

This systematic review found strong evidence in favor of using cfDNA as a prognostic tool in relation to mortality in trauma patients admitted to the ICU. We found that cfDNA can be used as a prognostic tool for the outcome of post-traumatic complications and the severity of trauma to a moderate extent.

With regard to linking cfDNA levels to clinical outcomes such as mortality and complications, we found that all of the included studies that presented survival data concluded that higher levels of cfDNA were significantly correlated to a higher mortality rate. It seems that cfDNA levels measured even at the earliest stages of ICU admission were able to predict a higher mortality rate [14, 31, 36, 39]. This finding was confirmed in 108 patients with sepsis [7]. Likewise, increasing levels of nDNA and mtDNA were significantly associated with increasing mortality [30, 31]. With regard to associating cfDNA levels with development of complications, the results were not conclusive. One study found that patients with high initial cfDNA and elevated levels of cfDNA on days 7 to 10 after ICU admission were significantly associated with sepsis [15]. Similar results were found in a study on a mixed population of ICU patients where cfDNA was measured on ICU admission, which found that cfDNA was higher in patients developing sepsis (*p* = 0.03) and in nonsurviving patients (*p* > 0.001) [40]. However, another prospective study on 160 consecutive ICU patients could not confirm these results [8], which may be due to the cfDNA processing. Interestingly, regarding the prognostic capabilities of cfDNA, it was found that cfDNA levels were superior to classification systems such as multiple organ dysfunction syndrome (MODS) regarding complications or APACHE II scores regarding mortality [41].

This systematic review has limitations as well as strengths. A strength of this review is that all of the included studies calculated severity score analysis of the trauma patients, and that the cfDNA levels were correlated to these scores. In general, high severity scores correlated to higher levels of cfDNA. A limitation is that even though we searched widely in four databases, the result of our search strategy may have been subject to publications bias, which we were not able to adjust for. Another limitation was that the patient populations differed to some extent between the included studies, which rendered it impossible to perform meta-analysis on the outcomes. No information regarding treatment strategies or regimes in the respective hospitals were available, which could lead to differences in outcome based on different treatments. Heterogeneity in cfDNA sampling and processing were found, which made direct comparison of the results difficult. Only one study reported on bacterial DNA analysis.

An interesting aspect of mtDNA is the hypothesis of mtDNA acting as a DAMP. In the light of the study by Xiao and collegues [42], where circulating leukocyte transcriptome after severe trauma and burn injury was measured, it could be argued that mtDNA is a cornerstone of the immune reaction. They found that following severe trauma and burn injury a global reprioritization occurs in >80% of the cellular functions and pathways, a phenomenon they call a “genomic storm”. The formation of neutrophil extracellular traps by mtDNA has also been proposed as another factor in immune reaction following trauma [43]. Another study where nDNA and mtDNA was associated with immune function was the study by Timmermanns et al. [44], who found that nDNA but not mtDNA correlated negatively with human leukocyte antigen (HLA-DR) expression, suggesting that nDNA could possibly function as a DAMP.

With regard to the future of cfDNA research in the trauma field it is of utmost importance that the correct methods of cfDNA sampling and processing are used so that cfDNA is correctly analyzed. It could be argued that cfDNA should be measured pre- and postsurgery as some of the same mechanics comes into action during surgery as in trauma. Ren et al. found that two patients who repeatedly underwent surgery had increased cfDNA levels after each surgery [37]. This aspect of cfDNA could be very interesting if the same prediction and prognostic pattern could be observed in patients undergoing surgery. cfDNA could also be viewed as a tool in identifying trauma patients who need immediate treatment.

## Conclusion

This systematic review provides an overview of the possible benefits of measuring cfDNA in relation to mortality, severity of trauma, and post-traumatic complications in trauma patients in the ICU. Moreover, it illustrates important aspects of DNA sampling and processing that should be considered when implementing cfDNA as measurement of injury severity. Finally, it encourages further research in this area with the aforementioned guidelines for DNA processing. The value of cfDNA, nDNA, and mtDNA should be investigated in patients undergoing major elective surgery.

## Additional files

Additional file 1: cfDNA measurement. CfDNA assessment checkpoints. (DOCX 14 kb)
Additional file 2: CfDNA measurement results. Results of cfDNA assessment checkpoints for each study. One point was given for if circulating DNA was analyzed in plasma. One point was given if blood was collected in either an EDTA tube or cell-free DNA tube. One point was given if blood was processed before 4 h. One point was given if blood was centrifuged one or more times. One point was given if blood was frozen at –80 °C or –20 °C depending on whether cfDNA analysis was based on specific sequence or cfDNA quantification, respectively. *GAPDH* glyceraldehyde 3-phosphate dehydrogenase, *NADH* nicotinamide adenine dinucleotide. (DOCX 25 kb)
Additional file 3: Risk of bias assessment. Results of risk of bias assessment for each study. (DOCX 18 kb)
Additional file 4: Simplified SIGN methodology checklist of COHORT studies. Risk of bias assessment checklist. (DOCX 17 kb)

## Abbreviations

AIS: Abbreviated injury scale
ALI: Acute lung injury
APACHE: Acute Physiology and Chronic Health Evaluation
ARDS: Acute respiratory distress syndrome
cfDNA: Cell-free DNA
DAMP: Damage-associated molecular pattern
GCS: Glasgow Coma Scale
ICU: Intensive care unit
ISS: Injury Severity Score
MOF: Multi-organ failure
mtDNA: Circulating mitochondrial DNA
nDNA: Circulating nuclear DNA
NPV: Negative predictive value
SIRS: Systemic inflammatory response syndrome
SOFA: Sepsis-related Organ Failure Assessment
TBI: Traumatic brain injury
