# Supplementary material for: The role of total cell-free DNA in predicting outcomes among trauma patients in the intensive care unit: a systematic review
Source: Crit Care. 2017 Jan 24;21:14. doi: 10.1186/s13054-016-1578-9 (PMC5260039; doi:10.1186/s13054-016-1578-9)
Supplement: Additional file 1: — cfDNA measurement. CfDNA assessment checkpoints. (DOCX 14 kb) [file 13054_2016_1578_MOESM1_ESM.docx]

Additional file 1: CfDNA measurement

1. Plasma or serum(1 or 0)
2. EDTA or cell-free DNA tubes(both are 1, others are 0)
3. Blood must be processed before 4h
4. 1, preferable 2 centrifugations
5. Preferable -80 degree freeze if measurement is by specific sequence, or -20 up to three months if quantification of cfDNA
