# Supplementary material for: The role of total cell-free DNA in predicting outcomes among trauma patients in the intensive care unit: a systematic review
Source: Crit Care. 2017 Jan 24;21:14. doi: 10.1186/s13054-016-1578-9 (PMC5260039; doi:10.1186/s13054-016-1578-9)
Supplement: Additional file 2: — CfDNA measurement results. Results of cfDNA assessment checkpoints for each study. One point was given for if circulating DNA was analyzed in plasma. One point was given if blood was collected in either an EDTA tube or cell-free DNA tube. One point was given if blood was processed before 4 h. One point was given if blood was centrifuged one or more times. One point was given if blood was frozen at –80 °C or –20 °C depending on whether cfDNA analysis was based on specific sequence or cfDNA quantification, respectively. GAPDH glyceraldehyde 3-phosphate dehydrogenase, NADH nicotinamide adenine dinucleotide. (DOCX 25 kb) [file 13054_2016_1578_MOESM2_ESM.docx]

| Additional file 2: CfDNA measurement results | |  |  |  |  |  |  |
| --- | --- | --- | --- | --- | --- | --- | --- |
| Study | 1^A^ | 2^B^ | 3^C^ | 4^D^ | 5^E^ | cfDNA analyzing method | Total |
| Non-specific trauma |  |  |  |  |  |  |  |
| McIlroy et al (2014) | 1 | 1 | 0(not described) | 1 | 1 | GAPDH | 4 |
| Lo et al (2000) | 1 | 1 | 0(not described) | 1 | 0(not described) | Beta-globin | 3 |
| Lam et al (study 1) (2003) | 1 | 0 | 0(not described) | 1 | 1 | Beta-globin | 3 |
| Lam et al (study 2) (2003) | 1 | 1 | 0(not described) | 1 | 1 | Beta-globin | 4 |
| Ren et al (2013) | 1 | 0(not described) | 1 | 1 | 1 | Beta-globin | 4 |
| Margraf et al (2008) | 1 | 1 | 1 | 1 | 1 | Fluorescent method | 5 |
| Wijeratne et al (2004) | 1 | 1 | 1 | 1 | 1 | Beta-globin | 5 |
| Yamanouchi et al(2013) | 1 | 0(not described) | 0(not described) | 1 | 0(not described) | NADH dehydrogenase | 2 |
| Lam et al (2004) | 1 | 1 | 0(not described) | 1 | 1 | Specific sequence | 4 |
| Gu et al(2013) | 1 | 1 | 0(not described) | 1 | 1 | Specific sequence | 4 |
| Traumatic brain injury |  |  |  |  |  |  |  |
| Yurgel VC et al(2007) | 1 | 0 | 0(not described) | 1 | 0 | Beta-globin | 2 |
| Shaked et al (2014) | 0 | 0 |  |  |  | Fluorescent method | ? |
| Filho et al(2014) | 1 | 1 | 1 | 1 | 0 | Beta-globin | 4 |
| Macher et al (2012) | 0 | 0 | 1 | 1 | 0 | Beta-globin | 2 |
| Wang et al (2014) | 1 | 1 | 1 | 1 | 0 | Beta-globin | 4 |

Additional file 2 GAPDH: Glyceraldehyde 3-phosphate dehydrogenase. NADH: Nicotinamide adenine dinucleotide. A: One point was given for if circulating DNA was analyzed in plasma. B: One point was given if blood was collected in either an EDTA tube or cell-free DNA tube. C: One point was given if blood was processed before 4 hours. D: One point was given if blood was centrifuged one or more times. E: One point was given if blood was frozen in -80^o^ C or -20^o^ C depending on whether cfDNA analysis was based on specific sequence or cfDNA quantification respectively.
