# Supplementary material for: The role of total cell-free DNA in predicting outcomes among trauma patients in the intensive care unit: a systematic review
Source: Crit Care. 2017 Jan 24;21:14. doi: 10.1186/s13054-016-1578-9 (PMC5260039; doi:10.1186/s13054-016-1578-9)
Supplement: Additional file 3: — Risk of bias assessment. Results of risk of bias assessment for each study. (DOCX 18 kb) [file 13054_2016_1578_MOESM3_ESM.docx]

| Additional file 3: Risk of bias assessment |  |  |  |  |  |  |  |  |  |  |
| --- | --- | --- | --- | --- | --- | --- | --- | --- | --- | --- |
| Article/checklist |  | 1.2 | 1.4 | 1.7 | 1.8 | 1.10 | 1.11 | 1.12 | 1.13 | Final |
| Traumatic brain injury |  |  |  |  |  |  |  |  |  |  |
| Yurgel VC et al(2007) |  | No | NA | Yes | No | Yes | NA | Yes | Yes | 6 |
| Shaked et al (2014) |  | Yes | NA | Yes | Yes | Yes | NA | NA | No | 7 |
| Filho et al(2014) |  | Yes | NA | Yes | No | Yes | NA | NA | No | 6 |
| Wang et al(2014) |  | Yes | NA | Yes | Yes | Yes | NA | Yes | Yes | 8 |
| Macher et al(2012) |  | Yes | NA | Yes | No | Yes | Yes | Yes | No | 6 |
| Non-specific Trauma |  |  |  |  |  |  |  |  |  |  |
| Wijeratne et al(2004) |  | No | NA | Yes | No | Yes | Yes | NA | No | 5 |
| Margraf et al(2008) |  | Yes | NA | Yes | No | Yes | Yes | NA | No | 6 |
| Ren et al(2013) |  | Yes | NA | Yes | No | Yes | Yes | Yes | No | 6 |
| McIlroy et al(2014) |  | Yes | NA | Yes | No | Yes | Yes | Yes | No | 6 |
| Lo et al (2000) |  | No | NA | Yes | No | Yes | Yes | NA | No | 6 |
| Lam et al (2003) | 1 | No | NA | Yes | Yes | Yes | Yes | Yes | No | 6 |
|  | 2 | No | NA | Yes | Yes | Yes | Yes | Yes( many lost in follow up | No | 6 |
| Yamanouchi et al(2013) |  | Yes | NA | Yes | No | Yes | Yes | Yes | No | 6 |
| Lam et al (2004) |  | Yes | NA | Yes | No | Yes | Yes | NA | No | 6 |
| Gu et al(2013) |  | Yes | NA | Yes | No | Yes | Yes | NA | Yes | 7 |
