# Supplementary material for: The role of total cell-free DNA in predicting outcomes among trauma patients in the intensive care unit: a systematic review
Source: Crit Care. 2017 Jan 24;21:14. doi: 10.1186/s13054-016-1578-9 (PMC5260039; doi:10.1186/s13054-016-1578-9)
Supplement: Additional file 4: — Simplified SIGN methodology checklist of COHORT studies. Risk of bias assessment checklist. (DOCX 17 kb) [file 13054_2016_1578_MOESM4_ESM.docx]

| ADDITIONAL FILE 4: Simplified SIGN methodology checklist of COHORT studies | | | | |
| --- | --- | --- | --- | --- |
|  | **Type of bias** | Applicability and consideration for our review | Final Checklist | |
| 1.1 |  | The study addresses an appropriate and clearly focused question: *Pre-selection process* | |  |
| 1.2 | **Selection** | *The groups studied are selected from source populations that are comparable in all aspects other than the factor under investigation.*  “YES” if random / consecutive selection of patients (with in- and exclusion criteria). | | X |
| 1.3 | **Selection** | *The study indicates how many of the people asked to take part in each of the groups being studied:* (Mostly) not applicable | |  |
| 1.4 | **Performance** | The likelihood that some subjects might have the outcome at the time of enrolment is assessed and taken into account in the analysis?  “YES” if delirium was (indirectly) assessed at time of enrolment. | | X |
| 1.5 | **Attrition** | *What percentage of individuals or clusters recruited into each arm of the study dropped out before the study was completed?*  Not applicable in most cohort studies due to relatively short follow-up. | |  |
| 1.6 | **Attrition** | *Comparison is made between full participants and those lost to follow-up, by exposure status.*  Not applicable in most cohort studies due to relatively short follow-up. | |  |
| 1.7 | **Detection** | The outcomes are clearly defined.  “YES” if endpoints or outcomes were clearly specified and used in analysis.  “NO” if patients have short follow-up or missing essential follow-up days. | | X |
| 1.8 | **Detection** | The assessment of outcome is made blind to exposure status.  “Not Applicable” when essential risk factors are possible to be blinded; e.g. age. “NO” if assessors could have been blinded for essential risk factors, but were not, e.g. cognitive / executive tests or before-after studies. | | X |
| 1.9 | **Detection** | *Where blinding is not possible, there is some recognition that knowledge of exposure status could have influenced the assessment of outcome.* | |  |
| 1.10 | **Detection** | *The measurement of exposure is reliable and if possible validated.*  *“*YES” if measurement / registration of (risk) factors was clearly defined. ”NO” if essential exposure is measured with definition or not validated*.* | | X |
| 1.11 | **Detection** | *Outcome assessment is validated and reliable.* Pre-selection: usage of DSM validated delirium assessment tool.  “YES” if the assessment tool was validated in the specific population; e.g. CAM-ICU instead of CAM in ventilated patients. | | X |
| 1.12 | **Detection** | *Exposure level or prognostic factor is assessed more than once.*  ‘Not applicable’ for most factors. “YES” if risk factors can be assessed at multiple time points, e.g. laboratory values and done. | | X |
| 1.13 /  1.14 | **Statistical**  **Analysis** | *The main potential confounders are identified and taken into account in the design and analysis. Have confidence intervals been provided?*  Pre-selection process; “YES” if essential confounders have been taken into account. “NO” if significant differences in characteristics have not been taken in to account or certain variables in the model could be consequence of delirium instead of cause. | | X |
| Adapted from: Scottish Intercollegiate Guidelines Network. Checklist 3 – Cohort studies. Version 2.0. Edinburgh 2012. http://www.sign.ac.uk/methodology/checklists.html | | | | |
